# Conserved gene expression in sperm reservoirs between birds and mammals in response to mating

**DOI:** 10.1186/s12864-017-3488-x

**Published:** 2017-01-18

**Authors:** Mohammad Atikuzzaman, Manuel Alvarez-Rodriguez, Alejandro Vicente Carrillo, Martin Johnsson, Dominic Wright, Heriberto Rodriguez-Martinez

**Affiliations:** 1 Department of Clinical and Experimental Medicine, Faculty of Medicine and Health Sciences, Campus HU/US, Developmental Biology, Linköping University, Lasarettsgatan 64/65, Lanken, floor 12, SE-581 85 Linköping, Sweden; 2 Department of Physics, Chemistry and Biology, Faculty of Science and Engineering, Linköping University, Linköping, Sweden

**Keywords:** Oviduct, Sperm reservoir, Microarray, Bioinformatics, Chicken, Pig

## Abstract

**Background:**

Spermatozoa are stored in the oviductal functional sperm reservoir in animals with internal fertilization, including zoologically distant classes such as pigs or poultry. They are held fertile in the reservoir for times ranging from a couple of days (in pigs), to several weeks (in chickens), before they are gradually released to fertilize the newly ovulated eggs. It is currently unknown whether females from these species share conserved mechanisms to tolerate such a lengthy presence of immunologically-foreign spermatozoa. Therefore, global gene expression was assessed using cDNA microarrays on tissue collected from the avian utero-vaginal junction (UVJ), and the porcine utero-tubal junction (UTJ) to determine expression changes after mating (entire semen deposition) or in vivo cloacal/cervical infusion of sperm-free seminal fluid (SF)/seminal plasma (SP).

**Results:**

In chickens, mating changed the expression of 303 genes and SF-infusion changed the expression of 931 genes, as compared to controls, with 68 genes being common to both treatments. In pigs, mating or SP-infusion changed the expressions of 1,722 and 1,148 genes, respectively, as compared to controls, while 592 genes were common to both treatments. The differentially expressed genes were significantly enriched for GO categories related to immune system functions (35.72-fold enrichment). The top 200 differentially expressed genes of each treatment in each animal class were analysed for gene ontology. In both pig and chicken, an excess of genes affecting local immune defence were activated, though frequently these were down-regulated. Similar genes were found in both the chicken and pig, either involved in pH-regulation (*SLC16A2, SLC4A9, SLC13A1, SLC35F1, ATP8B3, ATP13A3*) or immune-modulation (*IFIT5, IFI16, MMP27, ADAMTS3, MMP3, MMP12*).

**Conclusion:**

Despite being phylogenetically distant, chicken and pig appear to share some gene functions for the preservation of viable spermatozoa in the female reservoirs.

**Electronic supplementary material:**

The online version of this article (doi:10.1186/s12864-017-3488-x) contains supplementary material, which is available to authorized users.

## Background

Pigs and poultry are taxonomically distant animal species differing in reproductive anatomy and physiology. However, they both share internal fertilization, e.g. the capacity to store immunologically foreign spermatozoa in the female genital tract during the interval between mating and ovulation, this either being short (30–36 h in the case of the pig, with multiple ovulations over a 30 min period) or long (over several weeks, including recurrent daily ovulations as in modern, in the case of highly selected egg-layer poultry) [1–3]. The utero-tubal junction (UTJ) of the pig and the utero-vaginal junction (UVJ) of the chicken oviduct are analogous, a location where a subpopulation of spermatozoa are selectively stored post-mating, remaining alive and potentially fertile [1–3], before being gradually released for the fertilization of ovulated eggs [4–6]. In mice, the presence of spermatozoa in the oviduct leads to changes in gene expression, with upregulation of adrenomedullin and prostaglandin-endoperoxide synthase 2 transcripts [7]. Likewise, mating changes gene expression in the UVJ of the ancestral Red Junglefowl [8] as well as in an advanced intercross line (AIL, crossing between Red Junglefowl and White Leghorn chicken, [9]). Insemination has been reported as being capable of increasing mRNA expression of transforming growth factor beta (*TGFβs*) and TGFβ receptors (*TβRs*) but of decreasing mRNA expression of interleukin 1 beta (*IL1B*) and lipopolysaccharide induced TNF factor (*LITAF*) in the UVJ, which has also being implicated in the survival of sperm-storage tubuli (SST)-resident spermatozoa [10–12]. Studies in pigs have to date solely focused on the area of the oviduct where fertilization takes place [13–15]. Thus, in contrast to avian studies, trials in mammals have yet to examine the sperm reservoir areas.

Birds and pigs differ in internal genital tract anatomy, with chickens lacking accessory sexual glands. In contrast, the boar has a complete set of accessory glands whose concerted secretions form the seminal plasma; an heterogeneous fluid that accompanies the spermatozoa -embedded in the intraluminal cauda epididymis fluid- when emitted at ejaculation. In either animal class, semen is an immunologically foreign cell-suspension for the female, which should promptly elicit an immune response to eliminate it. It has been hypothesized that semen signals a genomic shift in the oviduct of the female that modulates the expression of genes involved in immune processes in both chickens [9–12, 16] and mammals [13–15], resulting in a state of immune tolerance during the lengthy storage of spermatozoa [17]. However, whether these divergent animal classes share a common mechanism is unclear.

Moreover, whether it is the entire semen (e.g. both the spermatozoa and the seminal fluid), the spermatozoa themselves or the cell-free seminal fluid that elicit such changes in gene expression in the sperm reservoirs is, to the best of our knowledge, poorly explored, with the exception of studies performed in Red Junglefowl [8] and mutant mice whose ejaculates were sperm-free [7]. The protein composition of seminal fluid has been extensively studied in chickens [18–20] and mammals [21–24] including the pig [25, 26]. In mammals, seminal plasma proteins are considered the most relevant for fertility [27–30], presumably owing to the induction of an initial but transient inflammation to clear microorganisms, superfluous gametes and proteins from the genital tract [26], followed by the induction of an immunological tolerance to paternal alloantigens via the expansion of regulatory T cells [22] following endometrial synthesis of toll-like receptor 4 (TLR4)-regulated cytokines and chemokines [24].

In chickens, the seminal fluid contains a few proteins classified as immune regulatory and/or defense such as gallinacin-9, ovotransferrin, serum albumin, thioredoxin, and peroxiredoxin-6 [18, 19]. Our own studies also indicate that the levels of immune-modulatory cytokines TGFβ2 and CXCL10 in the seminal fluid as well as the expression of the proteins Gallinacin-9 and Ig lambda chain C differed between low- and high egg-laying chickens [31], which might be related to sperm survival capacity in the female oviduct. This relationship depends on the function of the sperm reservoirs, which are highly correlated with fertility in both chickens [12] and pigs [2]. Modern domestic chickens and pigs are considered highly fertile. The modern layer poultry White Leghorn lays around 300 eggs per year, and a modern Swedish Landrace female pig produces over 26 live piglets per year. However, whether the oviduct sperm reservoirs respond to the entry of semen or SF/SP by a change in gene expression, and whether the response is similar between such different animal species, is yet to be tested.

In this study it is hypothesized that zoologically distant modern pigs and poultry, despite being selected for productivity (litter size or egg-laying rate, among other variables), share conserved mechanisms to tolerate the lengthy presence of immunologically-foreign spermatozoa in the oviduct sperm reservoirs. To test this hypothesis, microarray analyses on the functional sperm reservoir tissues of White Leghorn hens and Swedish Landrace sows were performed to identify gene expression changes in UVJ and UTJ after mating (entire semen deposition) or in vivo artificial infusion with sperm-free seminal fluid.

## Methods

### Experimental design

Gene expression analyses of the functional oviduct sperm reservoir (UVJ in chickens and UTJ in pigs) were performed in twelve modern White Leghorn breed female chickens (*Gallus gallus domestica,* Experiment 1) and twelve modern Swedish Landrace female pigs (*Sus scrofa domestica*, Experiment 2). The females of either species were allotted to one of three separate groups: a natural mating group (*n* = 4), where females (hen or sow) were mated to a single male each; sperm-free SF/SP inseminated group (*n* = 4) where females were artificially inseminated with pooled seminal fluid/plasma collected from the same males used for the mating group, and finally a control group (*n* = 4) of females that were neither mated nor inseminated. The oviduct reservoirs were collected post-mortem (UVJ, hens) or surgically (UTJ, sows) 24 h after treatments along with control animals. The tissues were either investigated for gene expression using custom-made chicken microarray (Roche NimbleGen, 12X 135 k array) or porcine gene chip microarray (Affymetrix, Inc. 3420 Central Expressway, Santa Clara, CA 95051, USA).

Animal husbandry and experimental handling were performed in compliance with the European Community (Directive 2010/63/EU) and current Swedish legislation (SJVFS 2015:24). Throughout all experiments, animals were handled carefully and in such a way as to avoid any unnecessary stress. The experiments were approved in advance by the “Regional Committee for Ethical Approval of Animal Experiments” (Linköpings Djurförsöksetiska nämnd) in Linköping, Sweden (permit no 75–12).

### Semen evaluation

Sperm concentration and motility were evaluated using a light microscope (Zeiss, Stockholm Sweden) equipped with a thermal plate (41 °C for chicken semen or 38 °C for pig semen), positive phase contrast optics (10x objective), a Charge Coupled Device (CCD) camera (UI-1540LE-M-HQ, Ueye, IDS Imaging Development Systems GmbH, Ubersulm, Germany), and the Qualisperm® Software (Biophos SA, Lausanne, Switzerland).

### Experiment 1

#### Experimental birds

A White Leghorn (WL) layer breed selected for high food conversion efficiency and commonly commercial bred for egg-production [32] was used. The details of the chicken rearing are described in Johnsson et al. 2012 [33]. Briefly, all chickens were kept separated by gender at the facilities of Linköping University (LiU). Food, water and perches were available *ad libitum* and chicken were held under controlled temperature and light regimes (12 h:12 h light/dark cycle) in 1–2 m^2^ pens depending on age for their first seven weeks.

#### Collection of semen, evaluation, mating and artificial insemination of seminal fluid

Chickens were subjected to semen collection and evaluation following the same procedure as our previous study [9]. Briefly, semen was collected by manual abdominal massage and was primarily extended with Dulbecco’s medium and examined in four replicates for sperm concentration and kinematics using a light microscope as described above. Only males yielding semen of high quality (sperm numbers and proportions of progressively motile spermatozoa, evaluated using the instrumentation detailed above) were selected for mating/insemination. Four hens were individually paired with males of proven fertility -using one male per hen (treatment 1). The collected semen from selected males was also subjected to centrifugation at 21,000 x g at 4 °C for 10 min. The supernatant (SF) was harvested and ejaculates pools (1 pool/male to make 4 individual pools) were made from four males used for the mating program. A 200 μl aliquot of pooled SF was inseminated into the cloaca using a plastic Pasteur pipette (Treatment 2). Four hens were left unmated or un-inseminated as controls.

#### Collection of UVJ

All hens (treatment 1 and 2) were euthanized by cervical dislocation followed by decapitation, 24 h after mating or insemination, along with the control hens. Immediately post-mortem, the oviduct segments were identified and dissected out under stereomicroscopy. The UVJ containing the SST was then collected using disposable razor blades, following classical descriptions [34] and snap-frozen in liquid nitrogen (LN_2_), prior to storage at −80 °C until further processed. A supplementary UVJ containing SST sample per mated hen was also fixed in 4% paraformaldehyde for histological confirmation of sperm presence in the SST-reservoirs. The confirmation of the presence of sperm was performed prior to the use of the UVJ tissues from mated or SF-infused or control hens in the microarray experiment.

#### Microarrays hybridization and scanning

Total RNA extraction (using Trizol), integrity evaluation, cDNA synthesis and custom-made microarray analysis (Roche NimbleGen Systems, Inc., Madison, WI, USA) were done following Atikuzzaman et al. 2015 [9]. A total of 12 microarrays (4 arrays per group) were run in this experiment.

### Experiment 2

#### Experimental pigs

Young mature boars (*n* = 5) of proven sperm quality (concentration, morphology and motility) and weaned sows (parity 1–3, *n* = 12) of the Swedish Landrace breed were recruited from a controlled breeding farm and individually kept in separate pens at the Translational Medicine Center (TMC/CBR-3) of Linköping University under controlled temperature and light regimes (12 h:12 h light/dark cycle). Pigs were fed with commercial feedstuff (Lantmännen, Stockholm, Sweden) according to national standards [35], provided with water *ad libitum* and with all animals receiving the same management.

#### Semen collection, evaluation and harvesting of seminal plasma

Semen was manually collected (gloved-hand method) weekly. Only ejaculates with at least 70% motile and 75% morphologically normal spermatozoa immediately after collection were used. Seminal plasma (SP) was harvested from the whole ejaculate after double centrifugation at 1,500xg for 10 min. The harvested crude-SP was kept at −20 °C, until use.

#### Detection of oestrus

The females were observed two times daily for pro-oestrus and oestrus behavioural signs while holding snout contact with a neighbouring boar, by the application of backpressure by experienced personnel. Animals that showed a standing oestrous reflex were considered to be in oestrus and were used in the experiments. Sows were randomly allotted to a control group (*n* = 4, unmated/non-inseminated), mated (Treatment 1, *n* = 4) or SP-inseminated (Treatment 2, *n* = 4).

#### Mating and insemination with seminal plasma

Sows were, on the first day of behavioral oestrus, either cervically inseminated (disposable AI-catheter, Minitüb, Munich, Germany) with 50 ml of Beltsville Thawing Solution (BTS, Control group); mated with a boar (Treatment 1 group) or artificially inseminated with 50 ml of SP (Treatment 2 group).

#### Collection of tissues

On the second day of standing oestrus (pre/periovulation) the sows were sedated by the administration of a mixture of 5 mg dexmedetomidine (Dexdomitor, Orion Pharma Animal Health, Sollentuna, Sweden) and 100 mg tiletamine hydrochloride/zolazepam hydrochloride (Zoletil vet, Virbac A/S, Kolding, Denmark) intramuscularly. General anesthesia was induced using sodium thiopental (Abbott Scandinavia AB, Solna, Sweden) 7 mg/kg body weight, intravenously, and was maintained with isoflurane (Baxter Medical AB, Kista, Sweden, 3.5-5%) administered via a tracheal cuffed tube by a close-circuit PVC-ventilator (Servo ventilator 900 D, SIEMENS-ELEMA AB, Solna, Sweden). Peripheral blood was collected (Vacutainer containing K2EDTA, Greiner Bio-One GmbH, Kremsmünster, Austria) centrifuged at 300 x g for 10 min at room temperature. The blood plasma was harvested and stored at −20 °C until analysed for oestradiol (E_2_) and progesterone (P_4_) concentrations. The left and right UTJ were exposed by mid-ventral incision. The complete UTJ of each side was removed immediately after clamping the irrigating blood vessels, being longitudinally divided into two equal pieces. One of the pieces was plunged in liquid nitrogen (LN_2_) and later stored at −80 °C while the other piece was fixed in 4% paraformaldehyde for histological confirmation of sperm presence. The confirmation of presence or absence of spermatozoa was done prior to use the UTJ tissues from mated or SP-infused or control sows for microarray experiment. The ovaries were photographed and the follicles visually counted. There was a mean of 22.30 ± 7.29 (mean ± standard deviation) follicles per sow, without significant differences between sow-groups.

#### Determinations of oestradiol and progesterone concentrations

Concentrations of oestradiol (E_2_) and progesterone (P_4_) were measured in individual blood plasma (50 μl) using porcine enzyme linked immune sorbent assay (ELISA) kits (Cat#MBS700342 and Cat#MBS703577, MyBiosource Inc., San Diego, CA, USA), after preparation of a standard curve for the individual hormones, following the manufacturer protocol. The optical density of each microplate well was determined using a microplate reader (TECAN, Sunrise GmbH, Grödig, Austria) set at 450 nm. Oestradiol concentrations (mean ± SD in pg/ml) were 376.50 ± 27.76 in controls, 349.10 ± 62.19 in mated and 294.20 ± 80.24 in SF-inseminated sows and those of progesterone (mean ± SD in ng/ml) were <0.68 ± 0.34 without significant differences between sow groups, confirming the animals were in pre/peri-ovulatory oestrus.

#### Microarrays hybridization and scanning

Total RNA was extracted using Trizol from UTJ samples and evaluated following the protocol used in Atikuzzaman et al. 2015 [9]. Equal amounts of total RNA (250 ng) from each UTJ were used to make cDNA using GeneChip® WT PLUS reagent kit (Affymetrix, Santa Clara, CA, USA) following the manufacturer protocol. Finally, 3.5 μg of fragmented and labelled single stranded complementary DNA (41 μl) was mixed with 109 μl of hybridization master mix to make a cocktail hybridization mix for a single reaction. The hybridization cocktail was then incubated first at 99 °C for 5 min, followed by a descent to 45 °C until loading on the array chip (Porcine gene 1.0 ST GeneChip® Cartidge Array, Affymetrix). A total of 130 μl of the cocktail hybridization mix was loaded into the array chip and they were incubated at 45 °C under rotation at 60 revolutions per minute for 16 h. The hybridized cartridge array chip was then unloaded and subjected to washing and staining using a GeneChip® Fluidics Station 450 (Affymetrix), to be finally scanned using the Affymetrix GeneChip® scanner GCS3000.

### Microarray data analysis and bioinformatics

The expression data of experiments 1 and 2 were processed using the Robust Multichip Average (RMA) normalization procedure, computing average expression values by background adjustment, quantile normalization between arrays, and summarization, as implemented in the oligo package of Bioconductor/DEVA Software (Roche NimbleGen, Inc, DEVA 1.2.1). The statistical analysis of the normalized gene expression data was performed using the open source RStudio package (RStudio, Inc. Version 0.98.507). Linear models using the empirical Bayes’ approach as implemented in the package ‘limma’ were used to calculate differentially expressed transcripts. Two different multiple testing corrections were applied. The first was a Benjamini-Hochberg False Discover Rate (FDR) correction [36], whilst the second was based on a permutation test. The permutation test was used in addition to the FDR test, given that a number of the custom probes used on the microarray (specifically those based on EST transcripts) were replicates of genes already represented on the array, thus the FDR threshold maybe overly restrictive. The permutation test was performed using the Limma package by randomising the class classifications, then calculating gene expression differentiation globally, before retaining the top 1% value. This was repeated 1,000 times, before the top 5% of permuted values were then used as an experiment-wide threshold (with this p-value corresponding to a nominal value of approximately *p* < 0.002). The redundant and uncharacterized transcripts were excluded from the list after both the multiple testing corrections to make a final list of differentially expressed genes. An enrichment analysis of these differentially expressed genes (both permuted and non-permuted) was performed via a statistical overrepresentation test for gene ontology (GO) biological process, comparing the total number of reference genes in the genome of *Gallus gallus* (15,789) and of *Sus scrofa* (21,398) using the Panther Classification System for GO [37]. The top 200 of these differentially expressed genes (100 upregulated and 100 downregulated genes based on the log fold change at *P* < 0.05) in both animal classes were selected for further bioinformatic analyses. The GOs of the top differentially expressed genes were analysed under the PANTHER GO-Slim Biological Process category. PANTHER extracted differentially expressed genes in both animal classes and those in the GO-term category of immune system process were then searched for functional pathways using the Kyoto Encyclopedia of Genes and Genomes (KEGG) database [38]. Additional molecular functions of these differentially expressed immune system process genes were extracted from the protein knowledge base of the UniProt Consortium [39].

## Results

### Both mating and insemination of sperm-free SF/SP elicited changes in gene expression in the sperm reservoirs of chicken and pig oviducts

**Fig. 1:**
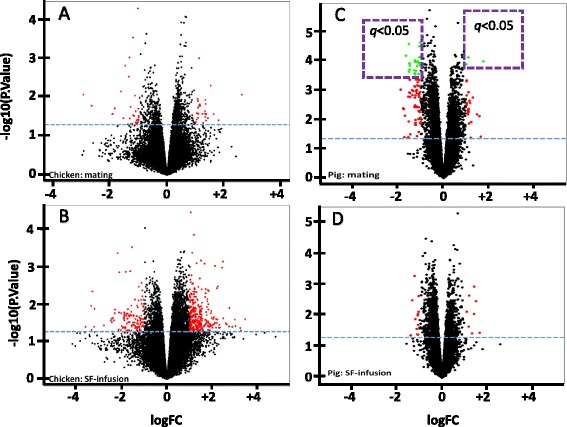
Volcano plots depicting differentially expressed probes for oviductal sperm reservoirs in chicken (UVJ) and pig (UTJ), following mating or sperm-free SF/SP-infusion. The x-axis represents the fold change and the y-axis represents the statistical significance (−log10 of P. value). Each of the oligonucleotide probes is represented by a single dot. The red dots represent log fold change > 1 or < −1 at *p*-value <0.05. The green dots represent log fold change >1 or < −1 at FDR adjusted *p*-value <0.05. The dots above the horizontal broken lines are probes that were differentially expressed at *p*-vaue <0.05. **a**) Comparison between mating (*n* = 4) and control (*n* = 4) group chicken, **b**) comparison between SF (*n* = 4) and control (*n* = 4) group chicken, **c**) comparison between mating (*n* = 4) and control (*n* = 4) group pigs and **d**) comparison between SF (*n* = 4) and control (*n* = 4) group pigs

Gene expression probes were calculated as pairwise comparisons (mating versus control and SF/SP-infusion versus control in chicken or pig) both with a FDR adjusted *p*-value < 0.05 and a permutation-adjusted p-value and were visualized by volcano plots (Fig. 1a-d). No gene probes were found to be differentially expressed at the FDR threshold in the chicken experiment, whilst in the pig this threshold led to the identification of 3 upregulated and 25 downregulated genes (Fig. 1c). Using the permutation threshold, a total of 41 (all condition comparisons), 18 (mating vs control) and 37 (SF-infusion vs control) genes were differentially expressed in the chicken experiment, while a total of 159 (all condition comparisons), 14 (mating vs control) and 198 (SP-infusion vs control) genes were differentially expressed in the pig experiment. In addition to these genes, differentially expressed with a nominal *p*-value of < 0.05 irrespective of multiple testing correction were considered suggestive in this study, particularly with regards to gene probes that had been identified in a previous experiment using mated and unmated chickens in a similar design [8, 9]. In the chicken UVJ, mating suggestively upregulated 504 gene probes and downregulated 324 gene probes (Fig. 1a). The sperm-free SF-infusion treatment upregulated 1,551 gene probes and downregulated 866 gene probes (Fig. 1b). In pig UTJ, mating upregulated 1,111 gene probes and downregulated 1,550 gene probes (Fig. 1c). Meanwhile, the SF-infusion upregulated 611 gene probes and downregulated 1,214 gene probes (Fig. 1d). Nevertheless, the gene probes in the volcano plots (Fig. 1) that did not represent characterized genes and were found to be redundant were excluded to make accurate final lists of differentially expressed genes in all comparisons for both animals. These lists are presented in the Additional file 1: Table S1, Additional file 2: Table S2, Additional file 3: Table S3 and Additional file 4: Table S4. In sum, mating in the chicken potentially changed the expression of up to 303 genes (189 genes were upregulated and 114 genes were downregulated) while SF-infusion changed the expression of 931 genes (513 genes were upregulated and 418 genes were downregulated), compared to controls. In the pig, mating elicited the differential expression of 1,722 genes (698 genes were upregulated and 1,024 genes were downregulated), while SF-infusion changed the expression of 1,148 genes (400 genes were upregulated and 748 genes were downregulated). These differentially expressed genes were then tested for a statistical overrepresentation of GO-biological processes involving immune functions. The immune function-related GO categories of these overrepresented genes (*P* < 0.05) are presented in Tables 1, 2, 3 and 4.

**Table 1:** Over- and under-represented mating-induced differentially expressed genes (*P* < 0.05) in the GO- biological process involving immune system function in chicken

GO-BP (immune function)	REF	DE (221)	EXPC	FE(+/−)	*P* value
CD8-positive, gamma-delta intraepithelial T cell differentiation (GO:0002305)	2	1	0.03	+35.72	2.76E-02
Gamma-delta intraepithelial T cell differentiation (GO:0002304)	2	1	0.03	+35.72	2.76E-02
Negative regulation of monocyte chemotaxis (GO:0090027)	2	1	0.03	+35.72	2.76E-02
Immunoglobulin secretion (GO:0048305)	2	1	0.03	+35.72	2.76E-02
T-helper 1 cell activation (GO:0035711)	2	1	0.03	+35.72	2.76E-02
Gamma-delta T cell activation (GO:0046629)	4	2	0.06	+35.72	1.50E-03
Chronic inflammatory response (GO:0002544)	3	1	0.04	+23.81	4.11E-02
Complement activation, lectin pathway (GO:0001867)	3	1	0.04	+23.81	4.11E-02
Negative regulation of immature T cell proliferation (GO:0033087)	3	1	0.04	+23.81	4.11E-02
Gamma-delta T cell differentiation (GO:0042492)	3	1	0.04	+23.81	4.11E-02
Negative regulation of T cell proliferation (GO:0042130)	24	2	0.34	+5.95	4.51E-02
B cell proliferation (GO:0042100)	24	2	0.34	+5.95	4.51E-02
Regulation of T cell proliferation (GO:0042129)	80	5	1.12	+4.47	5.69E-03
Positive regulation of T cell proliferation (GO:0042102)	52	3	0.73	+4.12	3.73E-02
Regulation of T cell activation (GO:0050863)	144	7	2.02	+3.47	4.50E-03
Positive regulation of T cell activation (GO:0050870)	91	4	1.27	+3.14	4.00E-02
Positive regulation of leukocyte cell-cell adhesion (GO:1903039)	95	4	1.33	+3.01	4.56E-02
Regulation of lymphocyte proliferation (GO:0050670)	120	5	1.68	+2.98	2.78E-02
Regulation of leukocyte proliferation (GO:0070663)	125	5	1.75	+2.86	3.23E-02
Regulation of leukocyte activation (GO:0002694)	249	8	3.49	+2.3	2.51E-02
Regulation of lymphocyte activation (GO:0051249)	219	7	3.07	+2.28	3.58E-02
Defence response (GO:0006952)	498	12	6.97	+1.72	4.92E-02

REF, Gallus gallus reference gene list (15789); DE, mating-induced differentially expressed genes (*P* < 0.05) in the UVJ; EXPC, expected number of genes in DE genes; FE, fold enrichment

**Table 2:** Over- and under-represented sperm-free SF-induced differentially expressed genes (*P* < 0.05) in the GO- biological process involving immune system function in chicken

GO-BP (immune function)	REF	DE (721)	EXPC	FE(+/−)	*P* value
Positive regulation of CD8-positive, alpha-beta cytotoxic T cell extravasation (GO:2000454)	1	1	0.05	+21.9	4.46E-02
Regulation of CD8-positive, alpha-beta cytotoxic T cell extravasation (GO:2000452)	1	1	0.05	+21.9	4.46E-02
Positive regulation of CD8-positive, alpha-beta T cell extravasation (GO:2000451)	1	1	0.05	+21.9	4.46E-02
Regulation of CD8-positive, alpha-beta T cell extravasation (GO:2000449)	1	1	0.05	+21.9	4.46E-02
Positive regulation of interleukin-15 production (GO:0032738)	1	1	0.05	+21.9	4.46E-02
Positive regulation of T cell extravasation (GO:2000409)	1	1	0.05	+21.9	4.46E-02
Regulation of T cell extravasation (GO:2000407)	1	1	0.05	+21.9	4.46E-02
Regulation of interleukin-15 production (GO:0032658)	1	1	0.05	+21.9	4.46E-02
Positive regulation of neutrophil apoptotic process (GO:0033031)	1	1	0.05	+21.9	4.46E-02
Positive regulation of isotype switching to IgA isotypes (GO:0048298)	1	1	0.05	+21.9	4.46E-02
T-helper 2 cell cytokine production (GO:0035745)	1	1	0.05	+21.9	4.46E-02
TIRAP-dependent toll-like receptor 4 signaling pathway (GO:0035665)	1	1	0.05	+21.9	4.46E-02
TIRAP-dependent toll-like receptor signaling pathway (GO:0035664)	1	1	0.05	+21.9	4.46E-02
Positive regulation of establishment of T cell polarity (GO:1903905)	1	1	0.05	+21.9	4.46E-02
Interleukin-8 biosynthetic process (GO:0042228)	1	1	0.05	+21.9	4.46E-02
Negative regulation of macrophage chemotaxis (GO:0010760)	4	2	0.18	+10.95	1.48E-02
Positive regulation of macrophage chemotaxis (GO:0010759)	5	2	0.23	+8.76	2.24E-02
Regulation of macrophage chemotaxis (GO:0010758)	11	4	0.5	+7.96	1.77E-03
Negative regulation of leukocyte chemotaxis (GO:0002689)	9	3	0.41	+7.3	8.50E-03
Positive regulation of macrophage differentiation (GO:0045651)	10	3	0.46	+6.57	1.13E-02
Regulation of macrophage differentiation (GO:0045649)	14	4	0.64	+6.26	4.17E-03
Negative regulation of alpha-beta T cell activation (GO:0046636)	12	3	0.55	+5.47	1.82E-02
Lymphocyte chemotaxis (GO:0048247)	14	3	0.64	+4.69	2.71E-02
Monocyte chemotaxis (GO:0002548)	16	3	0.73	+4.11	3.79E-02
Negative regulation of response to cytokine stimulus (GO:0060761)	27	4	1.23	+3.24	3.66E-02
Positive regulation of T cell proliferation (GO:0042102)	52	6	2.37	+2.53	3.39E-02
Regulation of leukocyte chemotaxis (GO:0002688)	57	6	2.6	+2.31	4.89E-02
Regulation of leukocyte migration (GO:0002685)	85	8	3.88	+2.06	4.39E-02

REF, Gallus gallus reference gene list (15789); DE, mating-induced differentially expressed genes (*P* < 0.05) in the UVJ; EXPC, expected number of genes in DE genes; FE, fold enrichment

**Table 3:** over- and under-represented mating-induced differentially expressed genes (*P* < 0.05) in the GO- biological process involving immune system function in pig

GO-BP (immune function)	REF	DE (1179)	EXPC	FE (+/−)	*P* value
Regulation of T cell mediated immune response to tumor cell (GO:0002840)	3	2	0.17	+12.1	1.22E-02
Regulation of macrophage apoptotic process (GO:2000109)	5	2	0.28	+7.26	3.16E-02
Interleukin-8 secretion (GO:0072606)	5	2	0.28	+7.26	3.16E-02
T-helper 17 cell differentiation (GO:0072539)	5	2	0.28	+7.26	3.16E-02
T-helper 17 type immune response (GO:0072538)	5	2	0.28	+7.26	3.16E-02
Positive regulation of mast cell chemotaxis (GO:0060754)	6	2	0.33	+6.05	4.39E-02
Toll-like receptor 4 signaling pathway (GO:0034142)	13	3	0.72	+4.19	3.61E-02
Positive regulation of monocyte chemotaxis (GO:0090026)	14	3	0.77	+3.89	4.33E-02
Positive regulation of leukocyte chemotaxis (GO:0002690)	59	7	3.25	+2.15	4.75E-02
Regulation of lymphocyte activation (GO:0051249)	239	20	13.17	+1.52	4.65E-02

REF, *Sus scrofa* reference gene list (21398); DE, mating-induced differentially expressed genes (*P* < 0.05) in the UTJ; EXPC, expected number of genes in DE genes; FE, fold enrichment

**Table 4:** Over- and under-represented sperm-free SP-induced differentially expressed genes (*P* < 0.05) in the GO- biological process involving immune system function in pig

GO-BP (immune function)	REF	DE (775)	EXPC	FE (+/−)	*P* value
Positive regulation of antigen processing and presentation of peptide antigen via MHC class II (GO:0002588)	1	1	0.04	+27.61	3.56E-02
Positive regulation of antigen processing and presentation of peptide or polysaccharide antigen via MHC class II (GO:0002582)	1	1	0.04	+27.61	3.56E-02
Cytokine secretion involved in immune response (GO:0002374)	1	1	0.04	+27.61	3.56E-02
B cell cytokine production (GO:0002368)	1	1	0.04	+27.61	3.56E-02
Positive regulation of IP-10 production (GO:0071660)	1	1	0.04	+27.61	3.56E-02
Regulation of IP-10 production (GO:0071658)	1	1	0.04	+27.61	3.56E-02
Positive regulation of B cell chemotaxis (GO:2000538)	1	1	0.04	+27.61	3.56E-02
Regulation of B cell chemotaxis (GO:2000537)	1	1	0.04	+27.61	3.56E-02
Positive regulation of mast cell activation by Fc-epsilon receptor signaling pathway (GO:0038097)	1	1	0.04	+27.61	3.56E-02
Tumor necrosis factor secretion (GO:1990774)	1	1	0.04	+27.61	3.56E-02
Isotype switching to IgG isotypes (GO:0048291)	1	1	0.04	+27.61	3.56E-02
T-helper 1 cell activation (GO:0035711)	1	1	0.04	+27.61	3.56E-02
Response to TNF agonist (GO:0061481)	1	1	0.04	+27.61	3.56E-02
Positive regulation of interleukin-1 alpha secretion (GO:0050717)	3	2	0.11	+18.41	5.49E-03
regulation of T cell mediated immune response to tumor cell (GO:0002840)	3	2	0.11	+18.41	5.49E-03
Positive regulation of interleukin-1 alpha production (GO:0032730)	4	2	0.14	+13.81	9.52E-03
Regulation of interleukin-1 alpha secretion (GO:0050705)	4	2	0.14	+13.81	9.52E-03
Toll-like receptor 2 signaling pathway (GO:0034134)	5	2	0.18	+11.04	1.45E-02
Regulation of interleukin-1 alpha production (GO:0032650)	5	2	0.18	+11.04	1.45E-02
Interleukin-10 production (GO:0032613)	5	2	0.18	+11.04	1.45E-02
Positive regulation of interleukin-1 secretion (GO:0050716)	11	4	0.4	+10.04	7.60E-04
Regulation of type I interferon-mediated signaling pathway (GO:0060338)	13	4	0.47	+8.5	1.40E-03
Positive regulation of interleukin-1 beta secretion (GO:0050718)	10	3	0.36	+8.28	6.03E-03
Positive regulation of type I interferon-mediated signaling pathway (GO:0060340)	7	2	0.25	+7.89	2.72E-02
Positive regulation of interleukin-1 production (GO:0032732)	14	4	0.51	+7.89	1.83E-03
Interleukin-8 production (GO:0032637)	7	2	0.25	+7.89	2.72E-02
Regulation of interleukin-1 beta secretion (GO:0050706)	14	4	0.51	+7.89	1.83E-03
Regulation of interleukin-1 secretion (GO:0050704)	18	5	0.65	+7.67	5.67E-04
Positive regulation of interleukin-10 production (GO:0032733)	19	5	0.69	+7.27	7.21E-04
Positive regulation of interleukin-8 secretion (GO:2000484)	8	2	0.29	+6.9	3.47E-02
Positive regulation of interleukin-1 beta production (GO:0032731)	12	3	0.43	+6.9	9.89E-03
Positive regulation of interferon-alpha production (GO:0032727)	12	3	0.43	+6.9	9.89E-03
Toll-like receptor 4 signaling pathway (GO:0034142)	13	3	0.47	+6.37	1.22E-02
Positive regulation of interleukin-6 secretion (GO:2000778)	13	3	0.47	+6.37	1.22E-02
Regulation of response to interferon-gamma (GO:0060330)	9	2	0.33	+6.14	4.28E-02
Inflammatory response to antigenic stimulus (GO:0002437)	14	3	0.51	+5.92	1.49E-02
Regulation of interferon-alpha production (GO:0032647)	14	3	0.51	+5.92	1.49E-02
Regulation of interleukin-10 production (GO:0032653)	26	5	0.94	+5.31	2.82E-03
Regulation of interleukin-1 beta production (GO:0032651)	27	5	0.98	+5.11	3.30E-03
Regulation of interleukin-1 production (GO:0032652)	34	6	1.23	+4.87	1.69E-03
MyD88-dependent toll-like receptor signaling pathway (GO:0002755)	17	3	0.62	+4.87	2.46E-02
Cellular response to interleukin-4 (GO:0071353)	18	3	0.65	+4.6	2.85E-02
Positive regulation of interferon-beta production (GO:0032728)	20	3	0.72	+4.14	3.71E-02
Positive regulation of type I interferon production (GO:0032481)	27	4	0.98	+4.09	1.76E-02
Somatic diversification of immune receptors via germline recombination within a single locus (GO:0002562)	28	4	1.01	+3.94	1.98E-02
Positive regulation of interleukin-6 production (GO:0032755)	42	6	1.52	+3.94	4.71E-03
Positive regulation of interleukin-8 production (GO:0032757)	30	4	1.09	+3.68	2.47E-02
Regulation of interferon-beta production (GO:0032648)	30	4	1.09	+3.68	2.47E-02
Regulation of type I interferon production (GO:0032479)	38	5	1.38	+3.63	1.33E-02
Toll-like receptor signaling pathway (GO:0002224)	40	5	1.45	+3.45	1.62E-02
Positive regulation of tumor necrosis factor production (GO:0032760)	34	4	1.23	+3.25	3.64E-02
Positive regulation of tumor necrosis factor superfamily cytokine production (GO:1903557)	35	4	1.27	+3.16	3.98E-02
Positive regulation of cytokine secretion (GO:0050715)	62	7	2.25	+3.12	8.19E-03
Positive regulation of innate immune response (GO:0045089)	90	10	3.26	+3.07	1.96E-03
Positive regulation of adaptive immune response (GO:0002821)	57	6	2.06	+2.91	1.88E-02
Regulation of cytokine-mediated signaling pathway (GO:0001959)	60	6	2.17	+2.76	2.35E-02
Regulation of interleukin-6 production (GO:0032675)	70	7	2.54	+2.76	1.50E-02
Regulation of response to cytokine stimulus (GO:0060759)	62	6	2.25	+2.67	2.69E-02
Positive regulation of adaptive immune response based on somatic recombination of immune receptors built from immunoglobulin superfamily domains (GO:0002824)	54	5	1.96	+2.56	4.85E-02
Regulation of innate immune response (GO:0045088)	154	14	5.58	+2.51	1.83E-03
Regulation of lymphocyte mediated immunity (GO:0002706)	82	7	2.97	+2.36	3.17E-02
Positive regulation of defense response (GO:0031349)	155	11	5.61	+1.96	2.82E-02
Regulation of immune effector process (GO:0002697)	260	18	9.42	+1.91	7.84E-03
Positive regulation of immune response (GO:0050778)	256	17	9.27	+1.83	1.39E-02
Regulation of inflammatory response (GO:0050727)	185	12	6.7	+1.79	4.03E-02
Regulation of leukocyte cell-cell adhesion (GO:1903037)	189	12	6.85	+1.75	4.59E-02
Regulation of defense response (GO:0031347)	394	25	14.27	+1.75	5.84E-03
Immune effector process (GO:0002252)	253	16	9.16	+1.75	2.46E-02
Regulation of immune response (GO:0050776)	398	25	14.41	+1.73	6.59E-03
Regulation of cytokine production (GO:0001817)	352	22	12.75	+1.73	1.09E-02
Innate immune response (GO:0045087)	280	17	10.14	+1.68	2.93E-02
Positive regulation of immune system process (GO:0002684)	481	29	17.42	+1.66	6.26E-03
Immune system development (GO:0002520)	470	28	17.02	+1.64	8.28E-03
Regulation of immune system process (GO:0002682)	785	45	28.43	+1.58	2.05E-03
Cytokine-mediated signaling pathway (GO:0019221)	295	4	10.68	- 0.37	1.81E-02

REF, *Sus scrofa* reference gene list (21398); DE, sperm-free SP-induced differentially expressed genes (*P* < 0.05) in the UTJ; EXPC, expected number of genes in DE genes; FE, fold enrichment

### Mating or SF-infusion changed the expression of treatment-specific genes as well as of a common subset of genes in the sperm reservoir

**Fig. 2:**
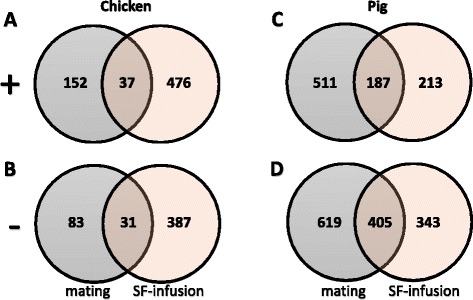
Venn diagrams of differentially expressed genes (*p* < 0.05) in the oviductal sperm reservoirs of chicken (UVJ) and pig (UTJ) after mating or SF/SP-infusion, compared to their controls: **a**) upregulated genes in the UVJ of chicken, **b**) downregulated genes in the UVJ of chicken, **c**) upregulated genes in UTJ of pigs, D) downregulated genes in UTJ of pigs

The number of differentially expressed genes in the chicken or pig is presented in a series of Venn diagrams, depicting treatment-specific/animal classes modified genes as well as genes that were considered common/conserved in the oviductal sperm reservoirs both after mating and SF-infusion (Fig. 2). Mating or in vivo SF-infusion changed the expression of a common subset of 68 genes in the chicken (37 genes were upregulated, while 31 genes were downregulated) and of 592 genes in the pig (187 upregulated and 405 downregulated).

### A subset of stimulus-responsive and immune system-process genes were differentially expressed in the sperm reservoirs after mating and SF-infusion; the expression pattern differed chicken and pigs after mating but not after in vivo SF-infusion

**Fig. 3:**
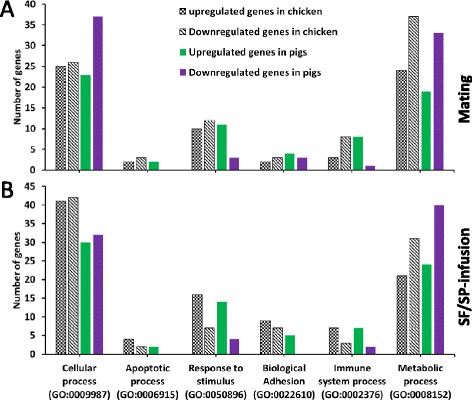
Biological process categories of top 200 differentially expressed genes (100 upregulated and 100 downregulated) selected from each comparison (mating versus control and SF/SP-infusion versus control in each animal class). The y-axis represents number of differentially expressed genes and the x-axis represents biological process categories analyzed by PANTHER gene ontology classification database. The black and white pattern columns represent differentially expressed genes in chicken while colored columns represent differentially expressed genes in pigs. **a**) Comparison between mating and control group of chicken and pig, **b**) comparison between SF/SP-infusion and control group of chicken and pig

To assess whether the same gene types were differentially regulated in both the chicken and pig, we took the top 200 differentially expressed genes (at a *p*-value <0.05, ranked in descending log fold change order of 100 upregulated and 100 downregulated), comparing mating or SF/SP-infusion classes with their respective controls in both animal classes, and performed a gene ontology analysis, whereby GO-categories were identified in each. This analysis revealed that a large subset of differentially expressed genes were involved in the GO term category of cellular and metabolic processes after mating or SF/SP-infusion (Fig. 3). The expression patterns (ratio of upregulated and downregulated genes) for genes involved in stimulus response and immune system processes differed between animal classes after mating (Fig. 3a), but not after SF-infusion (Fig. 3b). Mating changed the expression of stimulus-responsive genes in the chicken (10 upregulated while 12 were downregulated) and in the pig (11 upregulated while 3 downregulated) (Fig. 3a). Mating also changed the expression of immune-responsive genes in the chicken (3 upregulated, while 8 were downregulated) and in the pig (8 upregulated, 1 downregulated).

Each one of the differentially expressed immune system process genes were followed to map their pathway hierarchy in different categories and subcategories of the Kyoto Encyclopedia of Genes and Genomes (KEGG) database. The differentially expressed genes were mainly classified as the KEGG subcategory of immune system (10 genes), signal molecules and interaction (10 genes), signal transduction (8 genes) and endocrine system (3 genes). The pathways of these differentially expressed genes including UniProt molecular function as well as their possible role at the oviduct sperm reservoir after mating or SF/SP-infusion in chicken and pig are presented in Additional file 5: Table S5. A summary of these differentially expressed genes and their roles in immune defense is presented in Table 5.

**Table 5:** Differentially expressed immune-modulatory genes in the oviductal sperm reservoirs. For details see Additional file 5: Table S5

Treatment	Animals	Local Immune defense (LID) at oviduct	Differentially expressed genes in the oviductal primary sperm reservoirs
Mating	Chicken	Enhance LID at UVJ	*CCR9, TNFSF4, TFPI*
		Suppress LID at UVJ	*LHX3, MASP1, NPY6R, NRXN1, F2, PTK2, HSPA13, NELL1*
	Pig	Enhance LID at UTJ	*GZMK, LY96, CD36, LOC100513220, PDZD2, DPP4*
		Suppress LID at UTJ	*CSMD3, DRD2, SELL*
SF/SP-infusion	Chicken	Enhance LID at UVJ	*DLK2, CCL1, CCR4, LIF, NOX3, ASTL*
		Suppress LID at UVJ	*ADCYAP1R1, DLL4, BPIL3*
	Pig	Enhance LID at UTJ	*GPR116, F8, GZMK, PTK2B, LY96, SEMA6A*
		Suppress LID at UTJ	*TXNRD1, NOR-1*

### Mating or SF-infusion changed the expression of the same genes in the oviductal sperm reservoir of both chickens and pigs in some instances

The common functional category genes from the top 200 differentially expressed genes of each animal class were identified after mating or sperm-free SF-infusion (Table 6). In response to mating, the solute carrier family genes (*SLC16A2* and *SLC4A9* in chickens or *SLC13A1* and *SLC35F1* in pigs) were upregulated, while, the metalloproteinase group genes (*MMP27* in chickens or *ADAMTS3, MMP3* and *MMP12* in pigs) and the Tata box gene family (*TBX4* in chickens or *TBX20* in pigs) were downregulated. Among them, *SLC16A2* and *MMP3* were also found in the KEGG database of pathways. There were also common genes in both species that were differentially expressed after SF-infusion. However, none of them were found to have any KEGG pathways described to date.

**Table 6:** Differentially expressed genes (top 200) with similar function(s) between animal classes

Treatment	Changes	DE genes in SR		Category of the DE genes	UniProt Biological process and molecular function	KEGG category	KEGG subcategory	KEGG pathways
		Hens	Sows					
Mating	UP	*SLC16A2, SLC4A9*	*SLC13A1, SLC35F1*	solute carrier family	Transmembrane transporter activity	Organismal system	Endocrine system	Thyroid hormone signaling pathway
	DOWN	*MMP27*	*ADAMTS3, MMP3, MMP12*	metallopeptidase	Metallopeptidase activity, regulation of cell migration	Environmental information processing	Signal transduction	TNF signaling pathway
		*TBX4*	*TBX20*	T-box family	Transcription factor activity	-	-	-
SF/SP-infusion	UP	*CDH17, CDH19*	*CDH13*	cadherin family	Cell adhesion	-	-	-
		*IFIT5*	*IFI16*	interferon induced protein	Activation of innate immune response	-	-	-
		*LRRC18, LRRTM4, LUZP2*	*LRIG1*	leucine-rich repeat protein	Cytokine mediated signaling	-	-	-
		*PROM2*	*PROM1*	prominin	Cholesterol/cadherin/actinin binding	-	-	-
		*RGS4*	*RGS5*	regulator of GPCR	G-protein coupled receptor activity	-	-	-
		*SLC10A2, SLC4A9*	*SLC35F1, SLC7A7*	solute carrier family	Membrane transporter activity	-	-	-
	DOWN	*ATP8B3*	*ATP13A3*	ATPase	Membrane transporter activity	-	-	-
		*HOXB9, HOXD12*	*HOXA11B*	homeobox	Cell chemotaxis, transcription factor activity	-	-	-
		*TBX4*	*TBX20*	T-box family	Transcription factor activity	-	-	-

### Permutation tested differentially expressed genes are also overrepresented in the category of immune functions

Since our previous analysis based on the top 200 differentially expressed genes (*p < 0.05*) based on fold changes (largest to smallest order) may contain a number of false positives, we performed an additional analysis using just the significantly differentially expressed genes (as determined by a 5% experiment-wide permutation threshold). These results are presented in the Additional file 6: Table S6, Additional file 7: Table S7, Additional file 8: Table S8, Additional file 9: Table S9, Additional file 10: Table S10 and Additional file 11: Table S11. In summary, a few immune function categories were enriched, while the GO analysis failed to detect immune functional genes in the chicken (for multiple group comparisons see Additional file 6: Table S6 and for pairwise comparison see Additional file 7: Table S7 and Additional file 8: Table S8). In the pig, differentially expressed genes (for multiple group comparisons see Additional file 8: Table S8 and for pairwise comparison see Additional file 9: Table S9, Additional file 10: Table S10 and Additional file 11: Table S11) belonged to the immune function categories revealed by both enrichment and GO analysis. However, in both species the extent of over-representation of the immune function GO category was less when using only the genes that were significant with the permutation test, though in both cases fewer genes were used in the analyses.

## Discussion

In the present experiments the pattern of gene expression changes registered in the oviduct sperm reservoirs of zoologically distant modern, fertility-selected chicken and pigs were studied 24 h after mating or in vivo SF/SP-infusion. We find that both mating and sperm-free SF/SP insemination causes gene expression changes in the primary functional sperm reservoirs of hens and sows, as detected by cDNA microarray. One caveat with this is that two different microarrays have been used for these analyses (Affymetrix and Roche), which could lead to some variation in the results, despite the raw microarray data being normalized and processed similarly to provide a valid match of the pattern of gene expression changes between species.

### Mating and SF/SP-infusion modify gene expression in the oviductal sperm reservoirs in chicken and pigs

In the present study, the level of significance at an FDR adjusted *P* value < 0.05 excluded almost all genes, in fact all genes tested using domestic WL-chicken were excluded and only few genes in mated pigs were found to be significant. Although we are aware of the inclusion of several false positives (type I errors), we considered differentially expressed genes at a *p*-value of < 0.05 irrespective of FDR correction as suggestive, to compared the gene expression between commercial layer chickens and high fertility. Interestingly, the present results using WL-chickens differ with our previous study based on an Advanced Intercross Line (AIL, an intercross between Red Junglefowl and White Leghorn chickens) using the same platform, where fifteen genes were differentially expressed after mating [9]. Even more interestingly, the ancestor Red Junglefowl showed a more than 50-fold stronger differential expression [8] in response to mating or sperm-free SF-infusion, as compared to the AIL [9] and the WL here reported. Considering all these results, we assume that selection for higher fertility, at least in the chicken, might have an effect on gene expression in the oviductal sperm reservoirs after mating or artificial fertilization using a sperm-free SF-infusion. Consequently, we consider it possible that domestication and the selection for higher fertility has made the domestic hen oviduct less responsive to antigenic spermatozoa and seminal fluid. We cannot assume the same is happening in the pig, since we have not compared the modern pig with wild boar (*Sus scrofa*) under the same experimental conditions (mating or SP-infusion with controls) nor do we have evidence of such comparative studies being performed elsewhere.

The current results show that both mating and SF/SP-infusion are separately capable of modifying gene expression in the sperm reservoir (Fig. 2). However, irrespective of either mating or SF/SP-infusion, the number of differentially expressed genes varies; in the chicken, a large subset of genes were upregulated and comparatively a smaller subset of genes were downregulated (compare Fig. 2a with b), while in pigs, a small subset of genes were upregulated and comparatively a larger subset of genes were downregulated (compare Fig. 2c with d). Again in the chicken, a larger subset of differentially expressed genes in the UVJ were responsive to SF-infusion (476 upregulated and 387 downregulated), while a comparatively smaller subset of differentially expressed genes responded to mating (upregulated 152 and downregulated 83). In contrast, the pig UTJ responded to mating with modifications of gene expression for a larger subset of differentially expressed genes (upregulated 511 and downregulated 619), while SP-infusion only modified a comparatively small subset of differentially expressed genes (upregulated 213 and downregulated 343). The results indicate mating and/or SF/SP-infusion are able to induce gene expression changes including a certain subset of genes common to both treatments, primarily in pigs although a small number were also present in chickens (see the number of common genes shown in the Venn diagrams in Fig. 2).

The presence of spermatozoa in the sperm reservoir changed gene expression in the UVJ of the chicken [9–11] and in the oviduct of mice [7], similar to our current results. One could argue that since mating is the combination of spermatozoa and SF the subset of gene expression changes by the sperm-free SF-infusion should not differ from those differentially expressed genes modified by mating. Differences in anatomical location of the functional sperm reservoirs between the species might have influenced the dissimilar results obtained with the SF. For instance, the UVJ is quite close to the site of semen (or SF) deposition, while in the pig the UTJ is more distant from the cervix. However, any fluid placed in the cervix of pigs during artificial insemination is propelled to the UTJ within minutes, by way of contractions of the myometrium [40], an effect that is increased when seminal plasma is used [41]. Seminal fluid, which sperm are transported in in while being deposited into the female genitalia, contains a complex mixture of biological molecules, some of them (TGF-β, spermadhesins, β-defensins etc.) adsorbed to the sperm surface [19, 21, 28, 42, 43], that can be carried up to the oviduct by uterine contractions, the latter influenced by other SF-components, including hormones. SP-spermadhesins can for instance be adsorbed to the plasma membrane and transported to the UTJ [44] or all the way up to the oocyte zona pellucida [45]. However, it is still unclear whether sperm-free-SF/SP is able to reach to the oviductal sperm reservoir post-infusion. It has been shown that small- to medium-size molecules (similar to those components of the SP) suspended in buffer can pass to the oviduct of the pig, after cervical insemination [46]. Male chicken seminal fluid contains proteins identified as participating in defence and immunity processes [18, 19], also observed in our previous unpublished results. Chicken semen expresses different types of β-defensins, apparently to protect spermatozoa from microbial damage [47]. As well, TGF-β isoforms known to coat the surface of human spermatozoa [42], elicit changes in the UVJ of turkey hens [10].

To the best of our knowledge, this is the first study reporting that sperm-free-SF modifies gene expression in the oviduct sperm reservoirs of both chickens and pigs, with certain gene expression changes common to either semen or sperm-free SF deposition. Such results reinforce previous findings in cervical cells [48] and uterus [24] where components of the SF play central roles, including peptides, proteins and even microRNAs [49]. Sperm-free SP has been reported as being necessary to increase the expression of genes mainly related to cytokine synthesis in the mouse uterus [24]. A similar study found, however, low gene expression changes in the oviduct of the very same species [7]. Interestingly, our present results showed that sperm-free SF/SP could be a central player for gene expression changes related to cytokine production in the sperm reservoir (Tables 2 and 4). On the other hand, mating-induced enrichment in this GO category is either absent in chickens UVJ (Table 1) or very low in pig UTJ (Table 3). These results suggest, in agreement with Schjenken et al. [24], that there are components in the SF/SP that modulate cytokine production in the female, including genomic changes.

### The local immune defence is modulated by either mating or SF-infusion

The shift, either induced by mating or by sperm-free SF-infusion, of genes belonging to the immune function category of GO biological process is statistically overrepresented when compared with the reference genome in both species (Tables 1, 2, 3 and 4). Interestingly, the insemination of sperm-free SF caused the highest overrepresentation of a larger number of immune system function categories in both species compared to mating, in either species. Within mating, however, the fold enrichment in the statistical overrepresentation was found to be highest in the chicken. Considering that mating implies that both SF/SP and spermatozoa are involved, the data suggest that the presence of spermatozoa potentially suppress the influence of components of the seminal fluid. Similar functions, albeit to a lesser extent, are also revealed by the bioinformatics analysis of the more stringent subset of differentially expressed genes significant at a 5% permutation threshold (Additional file 6: Tables S6, Additional file 7: Table S7, Additional file 8: Table S8, Additional file 9: Table S9, Additional file 10: Table S10 and Additional file 11: Table S11). Pathway analysis of the top 200 differentially expressed genes showed that most of these genes were involved in the GO category of cellular and metabolic processes (Fig. 3a-b) in both the chicken and the pig. This finding is consistent for post-mating studies in mice [7] and in a chicken AIL (Red Junglefowl x White Leghorn) [9]. The patterns (ratio between up and downregulated genes) of mating-induced differentially expressed genes in the GO term categories were similar between species with the exception of the immune system process and stimulus-responsive genes (Fig. 3a). However, the SF-infusion upregulated a larger subset of immune system process genes (7 genes in chicken and 7 genes in pigs) compared to the smaller subset of downregulated genes in this category (3 genes in chicken and 2 genes in pigs) (Fig. 3b). Immune system process genes are considered to be one of the central players in sperm survival in the oviduct sperm reservoirs. The bioinformatics investigation of our present data revealed that a large subset of differentially expressed genes are involved in the suppression of local immune defence in the sperm reservoir in the chicken after mating (Table 5). Our previous microarray study in the AIL-chicken, which has a moderate egg-laying capacity [50], showed that mating induced immune modulatory gene expression changes [9]. Das et al. 2009 [11] reported that immune modulatory TGFβ isoforms and their receptors are expressed in the UVJ of WL-hens in the presence of resident sperm. In contrast, mating-induced expression changes of immune system process genes in the UTJ of pigs were largely involved in immune activation. A microarray study in mice [7] reported that immune defence genes were also upregulated in the oviduct after mating. In all these studies, the interval between sperm deposition and the gene expression changes was restricted, covering the time spermatozoa were present in the sperm reservoir, and activation could thus be considered to play a role in the elimination of redundant spermatozoa and foreign proteins/pathogens, cleansing the internal genital tract for the descending embryos. In the chicken, where such events of internal embryo development do not exist, spermatozoa are present for weeks in the sperm reservoirs and the initial activation has to be rapidly changed to suppress the immune rejection of the foreign spermatozoa, thus protecting the sperm prior to transport to the site of fertilization. To what extent the sperm-free SF interplays with the above events remains to be explored, particularly in relation to which components signal the genomic shifts that we observed.

### Chicken and pig oviduct sperm reservoirs conserve common mechanisms of pH-regulation and immune-modulation

To assess the potential for overlap in genes within a similar functionality group potentially common between chicken and pig, the top 200 differentially expressed genes (100 upregulated and 100 downregulated genes) whose expression was modified either by mating or by SF-infusion in chicken or in pig, were compared. A total of 30 genes of few functional categories were shared between chickens and pigs amongst the most differentially expressed (i.e. within the top 200) in each comparison (Table 6). The genes that were identified in the common functional categories for chickens and pigs from these top 200 differentially expressed genes play prominent functions in either species, such as pH regulation (cell membrane transporters-solute carrier family genes e.g. avian *SLC16A2*, *SLC4A9*, *SLC10A2* or porcine *SLC13A1*, *SLC35F1*, *SLC7A7* and ATPases genes e.g. avian *ATP8B3* or porcine *ATP13A3*) or immune-modulation (metallopeptidases genes e.g. avian MMP27 or porcine *ADAMTS3*, *MMP3*, *MMP12* and interferon induced protein related genes- e.g. avian *IFIT5* or porcine *IFI16*), confirming previous findings in the chicken using an AIL [9] and in mice [7] oviducts.

Spermatozoa are apparently quiescent while stored in the oviduct functional sperm reservoir, their motility increasing when leaving the reservoir [51–53]. Sperm motility is highly sensitive to pH and it is rapidly affected by changes in pH levels. In domestic poultry (chickens, quails and turkeys) and mammals (cows and pigs), *in vitro* studies revealed that sperm motility is highest at an alkaline pH and it is possible to alter them towards quiescence if they are exposed to a low pH [54, 55]. In chickens, pH values below 7.8 inhibit sperm motility, and at this level sperm motility remains low, while raising the pH value 0.2 units and higher provides vigorous sperm motility [53]. The pig cauda epididymis has a pH value around 6.5 with quiescent spermatozoa [56]; their motility becoming activated by exposure to high pH or increasing bicarbonate levels [55, 56]. The sperm reservoirs of the sow register lower pH levels (6.7) compared to the upper tubal segments where fertilization takes place (ampullary-isthmic junction: 7.5; ampulla: 8.3 [54]) adding circumstantial evidence to the suggestions that changes in pH from acidic to alkaline would also regulate sperm transfer to the fertilization site [57]. The genes of solute carrier family and ATPases are involved in pH regulation by exchanging protons, ions and HCO_3_^−^ between the intra and the extracellular space [58–61]. Regulation of sperm motility in the oviduct sperm reservoir of either species might, therefore, be controlled through modifications of the expression of these genes of the solute carrier family and ATPases, such as the ones reported here by mating and SF/SP-infusion.

The identified genes of immune modulatory function that were common between animal classes were either upregulated or downregulated by either mating or sperm-free SF/SP. Matrix metallopeptidase genes (*MMP27* in chicken, or their counterparts in pig *ADAMTS3, MMP3* and *MMP12*) were downregulated post-mating while genes controlling interferon-induced proteins (*IFIT5* in chicken, or *IFI16* in pig) were upregulated after SF/SP-infusion (see Table 6). Matrix metalloproteinases (MMPs) and interferon-induced proteins were previously detected in the oviduct of chickens [62], mice [63] and cows [64]. The previous reports suggested that matrix metalloproteinases are involved in immunomodulation [65]. The *MMP-27* gene is expressed in the CD163^+^/CD206^+^ M2 macrophages in the cycling human endometrium [66], *MMP-3* KO-mouse reduced neutrophil influx in immune-mediated lung injury [67] and macrophage number in atherosclerotic plagues [68]. The *MMP-12* KO-mouse also reduced neutrophil influx in immune-mediated lung injury [69], macrophage migration [70] and reduced active TNF-α release from macrophages [71]. Therefore, downregulation of these genes might have an immune-suppressive role in the oviductal sperm reservoir in either animal class hereby considered. However, SF-insemination upregulated the expression of *IFIT5* and *IFI16* that might play role in immune-activation in the sperm reservoir since these genes were reported to have potential roles in enhancing innate immune and inflammatory response [72, 73]. Interestingly, these genes (*IFIT5* and *IFI16*) were not upregulated post-mating in the sperm reservoir, and appeared to be suppressed by the presence of spermatozoa in either species, following our bioinformatics analysis of overrepresented immune function categories. This suggests these genes help create an immune-balanced physiological environment tailored for sperm survival. However, more research is necessary to expand upon such mechanisms.

## Conclusion

Chickens and pigs apparently share common functional genes that induce changes post-mating that influence mechanisms for pH-regulation. The upregulated genes are often found to be the solute carrier family genes (*SLC16A2, SLC4A9, SLC35F1* and *SLC35F1*), whilst matrix metalloproteinases (*MMP27, ADAMTS3, MMP3* and *MMP12*) are downregulated, indicating potentially conserved mechanisms govern fertility in these two species.

## Additional files

Additional file 1: Table S1. Differentially expressed genes in the UVJ of mated hens compared to control. (XLSX 42 kb)
Additional file 2: Table S2. Differentially expressed genes in the UVJ of sperm-free SF-inseminated hens compared to control. (XLSX 105 kb)
Additional file 3: Table S3. Differentially expressed genes in the UTJ of mated sows compared to control. (XLSX 205 kb)
Additional file 4: Table S4. Differentially expressed genes in the UTJ of sperm-free SP-inseminated sows compared to control. (XLSX 135 kb)
Additional file 5: Table S5. Differentially expressed immune system process genes in the oviductal sperm reservoirs of chicken and pigs. (DOCX 28 kb)
Additional file 6: Table S6. Differentially expressed genes (multiple condition comparisons) with permutation test in chicken. (XLSX 20 kb)
Additional file 7: Table S7. Differentially expressed genes (mating vs control) with permutation test in chicken. (XLSX 17 kb)
Additional file 8: Table S8. Differentially expressed genes (SF vs control) with permutation test in chicken. (XLSX 19 kb)
Additional file 9: Table S9. Differentially expressed genes (multiple group comparisons) with permutation test in chicken. (XLSX 39 kb)
Additional file 10: Table S10. Differentially expressed genes (mating vs control) with permutation test in pig. (XLSX 16 kb)
Additional file 11: Table S11. Differentially expressed genes (SP vs control) with permutation test in pig. (XLSX 40 kb)

## Abbreviations

cDNA: Complementary deoxyribonucleic acid
GO: Gene ontology
RNA: Ribonucleic acid
SF: Seminal fluid
SP: Seminal plasma
TLR4: Toll-like receptor 4
UTJ: Utero-tubal junction
UVJ: Utero-vaginal junction
