# Erratum to: Conserved gene expression in sperm reservoirs between birds and mammals in response to mating

**DOI:** 10.1186/s12864-017-3919-8

**Published:** 2017-07-25

**Authors:** Mohammad Atikuzzaman, Manuel Alvarez-Rodriguez, Alejandro Vicente-Carrillo, Martin Johnsson, Dominic Wright, Heriberto Rodriguez-Martinez

**Affiliations:** 1 Department of Clinical and Experimental Medicine, Faculty of Medicine and Health Sciences, Campus HU/US, Developmental Biology, Linköping University, Lasarettsgatan 64/65, Lanken, floor 12, SE-581 85 Linköping, Sweden; 2 Department of Physics, Chemistry and Biology, Faculty of Science and Engineering, Linköping University, Linköping, Sweden

## Erratum

After publication of the original article [1] the authors found that the spelling of author Alejandro Vicente-Carrillo’s surname was incorrect.

In the original article, the hyphen was missed between Vicente and Carrillo and was incorrectly spelt Vicente Carrillo, when the correct spelling was Vicente**-**Carrillo.

The original article has been corrected.
