# Supplementary material for: Conserved gene expression in sperm reservoirs between birds and mammals in response to mating
Source: BMC Genomics. 2017 Jan 18;18:98. doi: 10.1186/s12864-017-3488-x (PMC5242001; doi:10.1186/s12864-017-3488-x)
Supplement: Additional file 5: Table S5. — Differentially expressed immune system process genes in the oviductal sperm reservoirs of chicken and pigs. (DOCX 28 kb) [file 12864_2017_3488_MOESM5_ESM.docx]

Supplementary table S5. Differentially expressed immune system process genes in chicken and pigs analyzed by PANTHER gene ontology classification database involved in different molecular functions and pathways analyzed by UniProt and Kyoto Encyclopedia of Genes and Genomes.

|  | | **CHICKEN: mating vs control** | | | | | | | |
| --- | --- | --- | --- | --- | --- | --- | --- | --- | --- |
| **Gene symbol** | **Gene description** | | **logFC** | **Expression** | **UniProt GO-Biological process function** | **Possible roles in the UVJ** | **KEGG category** | **KEGG subcategory** | **KEGG pathways** |
| LHX3 | LIM homeobox 3 | | 0.41 | UP | Negative regulation of apoptotic process | Immune-suppression | None |  |  |
| CCR9 | chemokine (C-C motif) receptor 9 | | 0.48 | UP | C-C chemokine receptor activity, scavenger receptor activity | Immune-activation | Environmental Information Processing | Signaling molecules and interaction | Cytokine-cytokine receptor interaction |
|  |  | |  |  |  |  | Organismal Systems | Immune system | Intestinal immune network for IgA production |
|  |  | |  |  |  |  |  |  | Chemokine signaling pathway |
| TNFSF4 | tumor necrosis factor superfamily member 4 | | 0.5 | UP | Inflammatory response, positive regulation of cytokine production and t cell proliferation | Immune-activation | Environmental Information Processing | Signaling molecules and interaction | Cytokine-cytokine receptor interaction |
| MASP1 | mannan-binding lectin serine peptidase 1 (C4/C2 activating component of Ra-reactive factor) | | -0.91 | DOWN | complement activation | Immune-suppression | Organismal Systems | Immune system | Complement and coagulation cascades |
| NPY6R | neuropeptide Y receptor Y6 (pseudogene) | | -0.35 | DOWN | Cell surface receptor signaling | Immune-suppression | Environmental Information Processing | Signaling molecules and interaction | Neuroactive ligand-receptor interaction |
| NRXN1 | neurexin 1 | | -0.85 | DOWN | Cell adhesion | Immune-suppression | None |  |  |
| F2 | coagulation factor II, thrombin | | -0.83 | DOWN | Acute inflammatory response | Immune-suppression | Environmental Information Processing | Signaling molecules and interaction | Neuroactive ligand-receptor interaction |
| TFPI | tissue factor pathway inhibitor (lipoprotein-associated coagulation inhibitor) | | -0.47 | DOWN | Serine-type endopeptidase inhibitor activity | Immune-suppression | Organismal Systems | Immune system | Complement and coagulation cascades |
| PTK2 | protein tyrosine kinase 2 | | -0.56 | DOWN | Innate immune response | Immune-suppression | Environmental Information Processing | Signal transduction | ErbB signaling pathway |
|  |  | |  |  |  |  |  |  | VEGF signaling pathway |
|  |  | |  |  |  |  |  |  | PI3K-Akt signaling pathway |
|  |  | |  |  |  |  | Cellular Processes | Cell motility | Regulation of actin cytoskeleton |
|  |  | |  |  |  |  |  | Cellular commiunity | Focal adhesion |
|  |  | |  |  |  |  | Organismal Systems | Immune system | Leukocyte transendothelial migration |
|  |  | |  |  |  |  |  |  | Chemokine signaling pathway |
|  |  | |  |  |  |  |  | Development | Axon guidance |
| HSPA13 | heat shock protein family A (Hsp70) member 13 | | -0.48 | DOWN | ATP binding | Immune-suppression | None |  |  |
| NELL1 | NEL-like 1 (chicken) | | -1.02 | DOWN | Calcium ion binding | Immune-suppression | None |  |  |
| **PIG: mating vs control** | | | | | | | | | |
| **Gene symbol** | **Gene description** | | **logFC** | **Expression** | **UniProt GO-Biological process function** | **Possible role in the UTJ** | **KEGG category** | **KEGG subcategory** | **KEGG pathways** |
| CSMD3 | CUB and Sushi multiple domains 3 | | 0.86 | UP | None | Immune-suppression | None |  |  |
| GZMK | granzyme K (granzyme 3; tryptase II) | | 1 | UP | Serine-type endopeptidase activity | Immune-activation | None |  |  |
| DRD2 | dopamine receptor D2 | | 0.88 | UP | Negative regulation of innate immune response | Immune-suppression | Environmental Information Processing | Signal transduction | Rap1 signaling pathway |
|  |  | |  |  |  |  |  |  | cAMP signaling pathway |
|  |  | |  |  |  |  |  | Signaling molecules and interaction | Neuroactive ligand-receptor interaction |
|  |  | |  |  |  |  | Cellular Processes | Cellular commiunity | Gap junction |
|  |  | |  |  |  |  | Organismal Systems | Nervous system | Dopaminergic synapse |
| LY96 | lymphocyte antigen 96 | | 0.65 | UP | Positive regulation of tumor necrosis factor production, toll-like receptor siganling | Immune-activation | Environmental Information Processing | Signal transduction | NF-kappa B signaling pathway |
|  |  | |  |  |  |  | Organismal Systems | Immune system | Toll-like receptor signaling pathway |
| CD36 | CD36 molecule (thrombospondin receptor) | | 0.76 | UP | Apoptotic cell clearence and phagocytosis by macrophage | Immune-activation | Environmental Information Processing | Signal transduction | AMPK signaling pathway |
|  |  | |  |  |  |  |  | Signaling molecules and interaction | ECM-receptor interaction |
|  |  | |  |  |  |  | Cellular Processes | Transport and catabolism | Phagosome |
|  |  | |  |  |  |  | Organismal Systems | Immune system | Hematopoietic cell lineage |
|  |  | |  |  |  |  |  | Endocrine system | Adipocytokine signaling pathway |
|  |  | |  |  |  |  |  |  | PPAR signaling pathway |
|  |  | |  |  |  |  |  | Digestive system | Fat digestion and absorption |
| LOC100513220 | probable G-protein coupled receptor 83-like | | 1.14 | UP | G-protein coupled receptor signaling | - | None |  |  |
| PDZD2 | PDZ domain containing 2 | | 0.81 | UP | Cell adhesion | Immune-activation | None |  |  |
| DPP4 | dipeptidyl-peptidase 4 | | 0.87 | UP | T cell activation, cell adhesion | Immune-activation | Organismal Systems | Digestive system | Protein digestion and absorption |
| SELL | selectin L | | -1.88 | DOWN | Cell adhesion, response to ATP | Immune-suppression | Environmental Information Processing | Signaling molecules and interaction | Cell adhesion molecules (CAMs) |
|  | | **CHICKEN: SF vs control** | | | | | | | |
| **Gene symbol** | **Gene description** | | **logFC** | **Expression** | **UniProt GO-Biological process function** | **Possible role in the UVJ** | **KEGG category** | **KEGG subcategory** | **KEGG pathways** |
| ADCYAP1R1 | ADCYAP receptor type I | | 1.23 | UP | Cell surface receptor signaling | Immune-suppression | Environmental Information Processing | Signal transduction | cAMP signaling pathway |
|  |  | |  |  |  |  |  | Signaling molecules and interaction | Neuroactive ligand-receptor interaction |
|  |  | |  |  |  |  | Organismal Systems | Endocrine system | Insulin secretion |
|  |  | |  |  |  |  |  |  | Renin secretion |
|  |  | |  |  |  |  |  | Environmental adaptation | Circadian entrainment |
| DLK2 | delta-like 2 homolog (Drosophila) | | 1.88 | UP | Calcium ion binding, regulation of fat cell differentiation | Immune-activation | None |  |  |
| CCL1 | chemokine (C-C motif) ligand 1 | | 1.04 | UP | Inflammatory response, neutrophil chemotaxis, chemokine activity | Immune-activation | None |  |  |
| CCR4 | chemokine (C-C motif) receptor 4 | | 1.05 | UP | Inflammatory response, chemotaxis | Immune-activation | Environmental Information Processing | Signaling molecules and interaction | Cytokine-cytokine receptor interaction |
|  |  | |  |  |  |  | Organismal Systems | Immune system | Chemokine signaling pathway |
| LIF | leukemia inhibitory factor | | 1.1 | UP | Immune response | Immune-activation | Environmental Information Processing | Signal transduction | Jak-STAT signaling pathway |
|  |  | |  |  |  |  |  |  | TNF signaling pathway |
|  |  | |  |  |  |  |  | Signaling molecules and interaction | Cytokine-cytokine receptor interaction |
|  |  | |  |  |  |  | Cellular Processes | Cellular commiunity | Signaling pathways regulating pluripotency of stem cells |
| NOX3 | NADPH oxidase 3 | | 1.27 | UP | Oxidoreductase | Immune-activation | None |  |  |
| ASTL | astacin-like metallo-endopeptidase (M12 family) | | 1.46 | UP | Cell adhesion, metallopeptidase activity, prevention of polyspermy during fertilization | Immune-activation | None |  |  |
| COL25A1 | collagen, type XXV, alpha 1 | | -1.27 | DOWN | beta-amyloid and heparin binding | - | None |  |  |
| BPIL3 | bactericidal/permeability-increasing protein-like 3 | | -3.2 | DOWN | Lipid binding | Immune-activation | None |  |  |
| DLL4 | delta-like 4 (Drosophila) | | -0.82 | DOWN | T cell differentiation | Immune-suppression | None |  |  |
|  | | **PIG: SF vs control** | | | | | | | |
| **Gene symbol** | **Gene description** | | **logFC** | **Expression** | **UniProt GO-Biological process function** | **Possible role in the UTJ** | **KEGG category** | **KEGG subcategory** | **KEGG pathways** |
| GPR116 | G protein-coupled receptor 116 | | 0.59 | UP | Innate immune response, leukocyte migration | Immune-activation | None |  |  |
| F8 | coagulation factor VIII, procoagulant component | | 0.58 | UP | Oxidoreductase activity | Immune-activation | Organismal Systems | Immune system | Complement and coagulation cascades |
| GZMK | granzyme K (granzyme 3; tryptase II) | | 0.64 | UP | Serine-type endopeptidase activity | Immune-activation | None |  |  |
| PTK2B | PTK2B protein tyrosine kinase 2 beta | | 0.47 | UP | Innate immune response, chmokine mediated signaling | Immune-activation | Environmental Information Processing | Signal transduction | Calcium signaling pathway |
|  |  | |  |  |  |  |  |  | Phospholipase D signaling pathway |
|  |  | |  |  |  |  | Organismal Systems | Immune system | Natural killer cell mediated cytotoxicity |
|  |  | |  |  |  |  |  |  | Leukocyte transendothelial migration |
|  |  | |  |  |  |  |  |  | Chemokine signaling pathway |
|  |  | |  |  |  |  |  | Endocrine system | GnRH signaling pathway |
| LOC100736569 | membrane cofactor protein-like | | 0.52 | UP | None | - | None |  |  |
| LY96 | lymphocyte antigen 96 | | 0.58 | UP | Positive regulation of tumor necrosis factor production, toll-like receptor siganling | Immune-activation | Environmental Information Processing | Signal transduction | NF-kappa B signaling pathway |
|  |  | |  |  |  |  | Organismal Systems | Immune system | Toll-like receptor signaling pathway |
| SEMA6A | sema domain, transmembrane domain (TM), and cytoplasmic domain, (semaphorin) 6A | | 0.72 | UP | Chemotaxis | Immune-activation | None |  |  |
| NOR-1 | neuron-derived orphan receptor-1 alfa | | -0.72 | DOWN | Positive regulation of leukocyte apoptotic process and mast cell cytokine production, monocyte aggregation | Immune-activation | Metabolism | Biosynthesis of other secondary metabolites | Aflatoxin biosynthesis |
| TXNRD1 | thioredoxin reductase 1 | | -0.52 | DOWN | Thioredoxin-disulfide reductase activity, cell redox homeostasis | Immune-suppression | None |  |  |
